# Effects of Methylcobalamin on Mitochondrial Alterations in Schwann Cells Under Oxidative Stress

**DOI:** 10.3390/biomedicines13102565

**Published:** 2025-10-21

**Authors:** Qicheng Li, Shiyan Liu, Lu Zhang, Tianze Sun, Yuhui Kou

**Affiliations:** 1 Department of Trauma and Orthopedics, Peking University People’s Hospital, Beijing 100044, China; qicheng.li@pku.edu.cn (Q.L.); LorraineZ0827@163.com (L.Z.); suntianze1997@163.com (T.S.); 2 Key Laboratory of Trauma and Neural Regeneration, Peking University, Beijing 100044, China; 3 Department of Physiology, School of Basic Medical Sciences, Shenzhen University, Shenzhen 518060, China; 2100243061@email.szu.edu.cn; 4 National Center for Trauma Medicine, Beijing 100044, China

**Keywords:** methylcobalamin, mitochondria, oxidative stress, RNA-Seq, Schwann cells

## Abstract

**Background/Objectives**: Peripheral neuropathy (PN) triggers early oxidative stress, disrupting Schwann cell homeostasis. In this context, mitochondria serve as a primary source and vulnerable target of reactive oxygen species (ROS). Here, we investigated whether methylcobalamin (MeCbl) mitigates oxidative stress-induced mitochondrial dysfunction. **Methods**: RSC96 cells were exposed to H_2_O_2_ to model oxidative injury, then treated with MeCbl. Mitochondrial network integrity was evaluated using super-resolution imaging coupled with quantitative morphometric analysis. RNA-sequencing was performed to identify differentially expressed genes (DEGs) and enriched biological pathways. Additionally, a network-pharmacology approach was employed to intersect the predicted MeCbl targets with the transcriptomic signature. **Results**: MeCbl treatment alleviated H_2_O_2_-induced mitochondrial fragmentation, restoring the interconnected reticulum characterized by increased branch number, total area, and a reduction in punctate mitochondria. Transcriptome analyses revealed the reprogramming of stress-response pathways. The DEGs were significantly enriched in processes including mitochondrial organization and dynamics, redox homeostasis, protein quality control, and pro-survival signaling. Network pharmacology demonstrated convergence between the MeCbl targets and DEGs at core nodes governing mitochondrial quality control and antioxidant defense, thereby providing a mechanistic basis for the imaging phenotypes. **Conclusions**: MeCbl improved the mitochondrial structure and remodeled the stress-response pathways in Schwann cells under oxidative stress. By linking high-resolution organelle phenotypes to molecular networks, these findings support MeCbl as a rational adjunct to mitigate oxidative stress-driven peripheral neuropathy and identify an intervenable regulatory axis for future targeted therapies.

## 1. Introduction

Peripheral neuropathy (PN) triggers a cascade of cellular stress responses at the site of damage, with oxidative stress being the dominant and early event [1,2]. As the principal glia of the peripheral nervous system, Schwann cells govern key processes including Wallerian degeneration, debris clearance, axonal metabolic support, and the construction of regenerative conduits [3]. Excess ROS impair these critical functions by damaging lipids, proteins, and nucleic acids and by interfering with the redox-sensitive signaling pathway [4]. At the organelle level, mitochondria serve as both a source and target of ROS, creating a self-amplifying vicious cycle that impairs bioenergetic metabolism, calcium regulation, and cell fate determination [5]. Therefore, breaking this cycle in Schwann cells represents a rational therapeutic strategy for restoring a neuro-reparative microenvironment.

The mitochondria in Schwann cells form a dynamic network, whose morphological characteristics such as branching structure, length, and connectivity reflect the balance between fission and fusion as well as the efficiency of mitochondrial autophagy. Oxidative stress shifts this balance toward fragmentation, depolarization, and impaired oxidative phosphorylation. These deficits reduced ATP production, thereby compromising both myelin maintenance and metabolic coupling between the axon and glia [4,6]. In vitro experiments, treatment with H_2_O_2_ provides a controlled model of oxidative damage, facilitating quantitative assessment of the mitochondrial structure and function. High-resolution mitochondrial imaging combined with morphometric analysis directly evaluates organelle integrity. When combined with transcriptomic sequencing, this approach captures pathway-level perturbations in key processes such as metabolism, dynamics, unfolded protein response, and antioxidant defense.

Methylcobalamin (MeCbl), as the coenzyme form of vitamin B12, is a cofactor for methionine synthase [7]. This role is critical for maintaining one-carbon metabolism [8], methylation capacity [9], and redox homeostasis [10]. Beyond these classical biochemical functions, studies have reported that MeCbl can promote axonal growth, support myelin integrity, and protect the nervous system from oxidative damage [11,12]. Potential mechanisms for these protective effects include the reinforcement of glutathione-dependent detoxification, modulation of mitochondrial biogenesis factors, and stabilization of electron transport chain under stress conditions. However, the extent of MeCbl’s direct protective effect on mitochondrial integrity in Schwann cells under oxidative stress as well as the underling signaling pathways remain incompletely elucidated.

In this study, we employed RSC96 cells to investigate the effects of MeCbl on mitochondria under oxidative stress conditions. By integrating super-resolution mitochondrial imaging with quantitative morphometric analysis, we assessed whether MeCbl alleviates stress-induced mitochondrial fragmentation and functional decline. To elucidate the molecular mechanisms, we performed RNA sequencing to identify DEGs and enriched pathways in MeCbl-treated cells compared with oxidative stress-induced cells. Furthermore, we employed a network pharmacology approach to cross-validate the predicted MeCbl targets with transcriptomic profiles, thereby identifying convergent nodes and pathways that may mediate its protective effects. This integrated approach aims to elucidate how MeCbl remodels the mitochondrial structure and redox signaling pathways in Schwann cells, thereby providing therapeutic insights for oxidative stress-related peripheral neuropathology.

## 2. Materials and Methods

### 2.1. Reagents

Methylcobalamin was purchased from MedChemExpress (Monmouth Junction, NJ, USA). Hydrogen peroxide (H_2_O_2_) was obtained from Sigma-Aldrich (Saint-Louis, MO, USA). TRIzol was purchased from Invitrogen Life Technologies (Carlsbad, CA, USA). PKMITO was obtained from GENVIVO (Nanjing, China).

### 2.2. Cell Culture

The RSC96 cells were purchased from Pricella (Wuhan, China). Cells were cultured in high-glucose DMEM (Gibco, Grand Island, NY, USA) supplemented with 10% FBS (Gibco, Grand Island, NY, USA) and 1% penicillin-streptomycin (HyClone, Logan, UT, USA). Cells were maintained at 37 °C in a humidified incubator under 5% CO_2_. For subculturing, cells at 80–90% confluence were detached using 0.25% Trypsin-EDTA (Gibco, Grand Island, NY, USA). Cells between passages 5 and 10 were used for all experiments. For subsequent assays, cells were seeded into culture plates at the appropriate density and allowed to adhere for 24 h before treatment.

### 2.3. Chemical Treatment

RSC96 cells were exposed to 0.2% H_2_O_2_ for 4 h to induce oxidative stress upon reaching 90% confluence [13]. After H_2_O_2_ treatment, the culture medium was replaced with fresh DMEM, supplemented with or without 50 μM methylcobalamin, and cells were further incubated for 24 h. Subsequently, cells were collected for RNA extraction and mitochondrial imaging to assess the effects of methylcobalamin against oxidative stress.

### 2.4. Mitochondrial Imaging and Analysis

RSC96 cells were plated onto PDL-coated 35 mm glass-bottom dishes and maintained under standard culture conditions. For mitochondrial labeling, cells were incubated with 250 nM PK Mito Red in DMEM at 37 °C for 15 min. Following staining, cells were washed twice with DMEM to remove residual dye. Subsequently, 2 mL of fresh DMEM was added, and live-cell imaging was performed using high-sensitivity structured illumination microscopy (HIS-SIM). The acquired images were quantitatively analyzed for mitochondrial morphological parameters including count, total area, and branches using the Mitochondria Analyzer plugin in ImageJ software (version 1.54p).

### 2.5. RNA Extraction, cDNA Library Preparation, and Sequencing

Total RNA was extracted from RSC96 cells with TRIzol reagent. The RNA concentration was determined using the Qubit Fluorometer (Invitrogen, Carlsbad, CA, USA). The RNA integrity was evaluated using an Agilent 2100 bioanalyzer (Agilent Technologies, Santa Clara, CA, USA). cDNA libraries were constructed using the NEB Next Ultra RNA Library Prep Kit (NEB, Ipswich, MA, USA) for Illumina. Poly(A) mRNA was isolated from 1 μg of total RNA using the NEB Next Poly(A) mRNA Magnetic Isolation Module Kit (NEB, Ipswich, MA, USA). The mRNA was fragmented into approximately 200 bp pieces, followed by first-strand cDNA synthesis using random hexamer primers and reverse transcriptase. The second-strand cDNA was synthesized using DNA polymerase I and RNase H. The cDNA fragments underwent end repair, followed by the addition of an “A” base and adapter ligation. The libraries were amplified through PCR, and the final products were purified. Library quality and concentration were assessed using the KAPA Library Quantification Kit (KAPA Biosystems, Cape Town, South Africa) and an Agilent 2100 Bioanalyzer. After RT-qPCR validation, paired-end sequencing (150 bp) was performed on an Illumina NovaSeq platform (Illumina, San Diego, CA, USA).

### 2.6. RNA-Seq Data Analysis

RNA-Seq data analysis was performed using the rat genome as the reference. Sequencing quality was assessed with FastQC (version 0.11.5), followed by the removal of low-quality reads using NGSQC (version 2.3.3). The clean data were mapped to the reference genome and assembled unique gene by Rsubread [14], and the expression level of genes was calculated by the featureCounts function. Between-group differential gene analysis was performed using DESeq2 (version 1.42.0) under the conditions of an adjusted *p* value ≤ 0.05 and |log2(Fold Change)| ≥ 0.25 [15].

### 2.7. GO and KEGG Analysis

To obtain insights into the change of phenotype, the GO enrichment analysis of DEGs was conducted using the clusterProfiler (version 4.12.6) R package, which corrects for gene length bias [16]. GO terms with an adjusted *p* value less than 0.05 were considered significantly enriched. The KEGG database provides insights into the high-level functions and utilities of biological systems, such as cells, organisms, and ecosystems, by interpreting molecular-level data, particularly large-scale datasets derived from genome sequencing and high-throughput experiments. The clusterProfiler R package was also used to identify KEGG pathways that were significantly enriched in DEGs.

### 2.8. Visualization of GO Gene Sets

To elucidate the relationship between gene expression and function, we depicted the significant pathways associated with GO terms of interest. Among the upregulated gene sets, our analysis highlighted pathways involved in the generation of precursor metabolites and energy, oxidative phosphorylation, cytoplasmic translation, and ribonucleoprotein biogenesis. Among the downregulated sets, we examined chromosome segregation and ATP-dependent activity, acting on DNA. The functional enrichments were visualized through the enrichplot package. The gene expression heatmap was completed through the pheatmap (version 1.0.12) package.

### 2.9. Protein–Protein Interaction (PPI) Network

To investigate the functional relationship among protein encoded by the enriched GO terms, we constructed PPI networks separately for the upregulated and downregulated gene sets. Official gene symbols were submitted to STRING for *Rattus norvegicus*, querying both physical and functional associations [17]. STRING interaction tables were imported into Cytoscape (version 3.10.3) for network analysis and figure preparation.

### 2.10. Network Pharmacology Validation

To validate the molecular mechanisms by which MeCbl regulates gene expression, we used the STITCH database (http://stitch.embl.de/, accessed on 28 August 2025) to identify the potential target genes. We first identified the upregulated and downregulated genes associated with MeCbl treatment using this resource. The retrieved gene list was subjected to GO enrichment analysis to investigate the biological functions and molecular pathways affected by the upregulated genes. The network pharmacology approach provided a comprehensive understanding of the molecular interactions and biological processes potentially influenced by MeCbl, facilitating further validation of its therapeutic effects.

### 2.11. Statistical Analysis

All analyses were performed in GraphPad Prism 8.0. Data were expressed as the mean ± SD. For comparisons between two independent groups, a two-tailed unpaired Student’s *t*-test was used. For comparisons among three groups, one-way ANOVA followed by Tukey’s multiple-comparisons test was performed. All tests were two-sided, exact *p* values are reported where feasible, and *p* < 0.05 was considered statistically significant.

## 3. Results

### 3.1. MeCbl Improved Mitochondrial Morphology of RSC96 Cells

**Figure 1:**
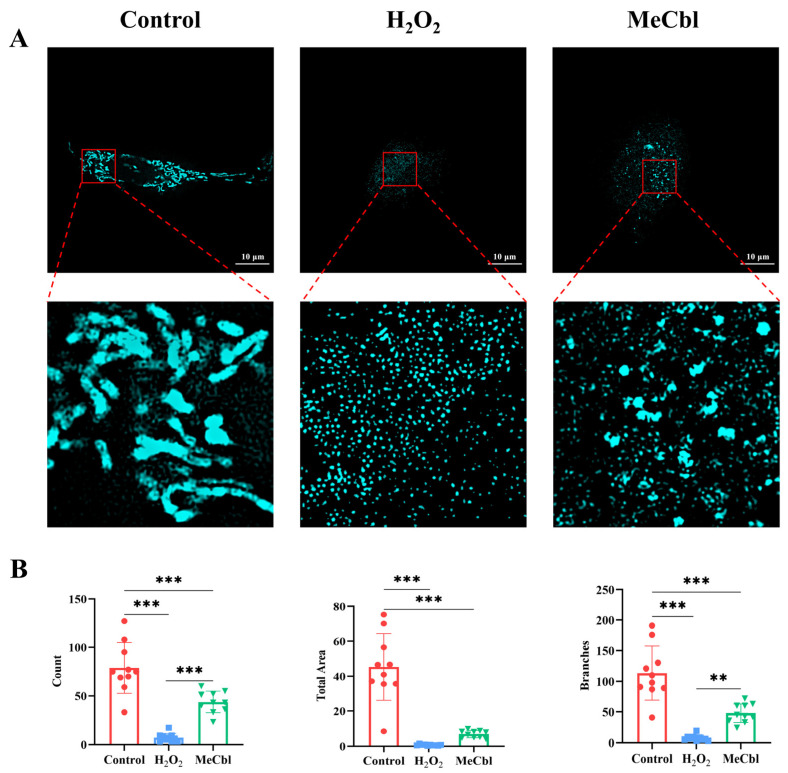
Mitochondrial changes of RSC96 cells. (**A**) Representative images of PK Mito Red-stained mitochondria in RSC96 cells. Scale bar = 10 μm. (**B**) Quantitative analysis of morphological changes of mitochondria in the control, H_2_O_2_, and MeCbl groups (*n* = 10, mean ± SD). ** *p* < 0.01, *** *p* < 0.001. A two-tailed Student’s *t*-test was used for comparisons between groups.

To assess the effects of oxidative stress and MeCbl on mitochondrial morphology, RSC96 cells were exposed to 0.2% H_2_O_2_ for 4 h and incubated in fresh DMEM with or without 50 μM MeCbl for an additional 24 h. Mitochondrial morphology was then observed in real-time using advanced imaging techniques (Supplementary Videos S1–S3). In the H_2_O_2_-treated group, mitochondria exhibited significant morphological changes including a marked reduction in size and a transition to a more spherical shape compared with the control group. In contrast, untreated RSC96 cells displayed typical, elongated, rod-shaped mitochondria (Figure 1A). Treatment with 50 μM MeCbl in H_2_O_2_-exposed cells led to a noticeable restoration of mitochondrial morphology. The mitochondrial count increased, and the proportion of branched structures was enhanced. Furthermore, the total mitochondrial area was partially restored, suggesting that MeCbl has a protective effect against oxidative stress-induced mitochondrial damage (Figure 1B). These findings indicate that MeCbl effectively mitigates oxidative stress-related mitochondrial dysfunction in RSC96 cells.

### 3.2. DEGs in the RSC96 Cells

**Figure 2:**
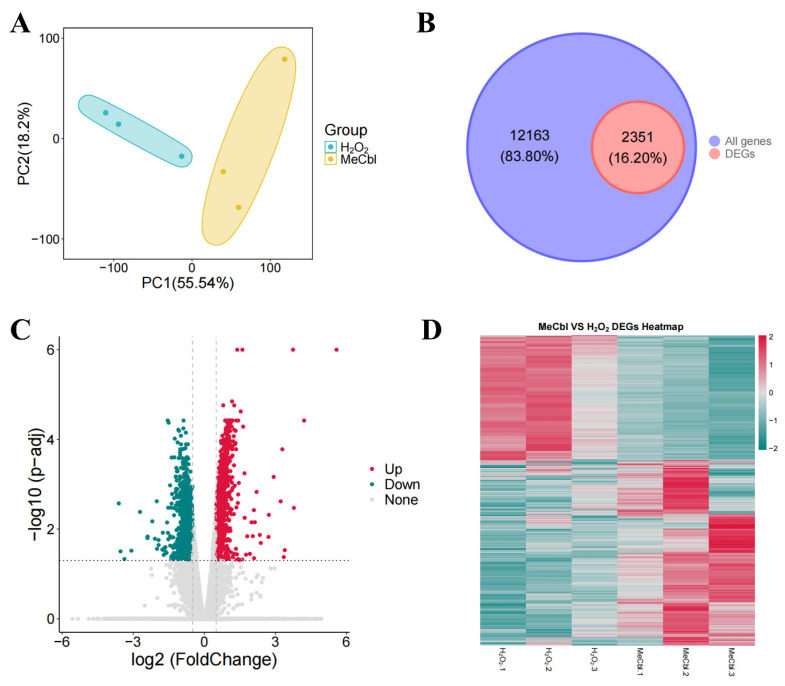
DEGs in the RSC96 cells treated with H_2_O_2_ and MeCbl. (**A**) Principal component analysis of the H_2_O_2_ and MeCbl groups. (**B**) Venn diagram of the percentage of DEGs in all genes. (**C**) Volcano plots showing upregulated and downregulated DEGs. (**D**) Heatmap of the H_2_O_2_ and MeCbl DEGs.

To explore the transcriptional alterations induced by MeCbl treatment under oxidative stress conditions, RNA sequencing was performed on RSC96 cells following 4 h of H_2_O_2_ exposure with or without subsequent 24 h incubation with 50 μM MeCbl. The results of the transcriptomic analysis are summarized in Figure 2. Principal component analysis (PCA) revealed clear separation between the H_2_O_2_-treated and MeCbl-treated groups, with principal components PC1 and PC2 accounting for 55.54% and 18.2% of the total variance, respectively (Figure 2A). The PCA results suggest that the sample features within two groups were similar, while the differences between the groups were significant. The Venn diagram showed that 2351 DEGs were identified, accounting for 16.2% of the total 12,163 genes analyzed (Figure 2B). Among these DEGs, 1377 genes were upregulated and 974 genes were downregulated, as visualized in the volcano plot (Figure 2C). This distribution indicates significant transcriptional changes in response to H_2_O_2_ and MeCbl treatment. Table 1 and Table 2 list the top ten up- and downregulated genes with the most significant differences and their functions, which mainly play a role in protein synthesis, energy metabolism, and chromosomal changes. To further investigate the expression patterns of these DEGs, we generated a heatmap that contrasts the gene expression profiles of the MeCbl-treated group against the H_2_O_2_ group (Figure 2D). These data suggest that MeCbl significantly modulates gene expression in response to oxidative stress.

### 3.3. GO Analysis of DEGs

**Figure 3:**
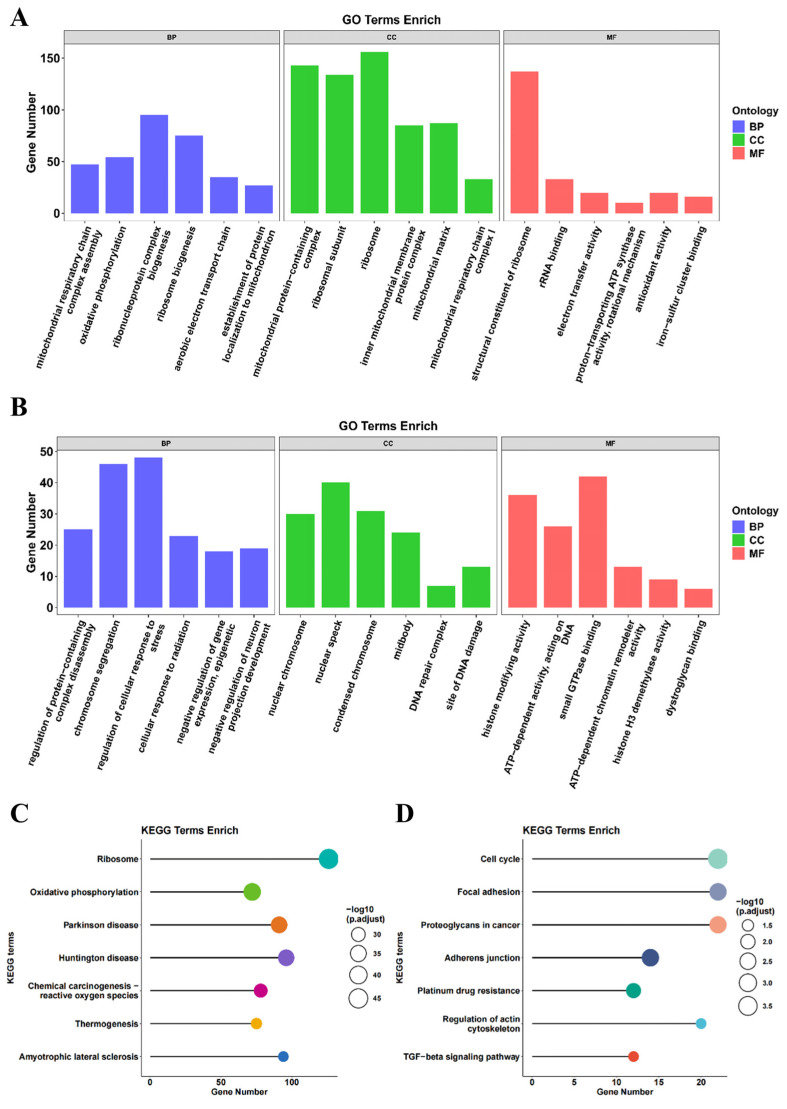
Functional analysis of DEGs by GO and KEGG classifications. (**A**,**B**) The GO enrichment analysis of representative up- and downregulated DEGs. (**C**,**D**) The KEGG enrichment analysis of representative up- and downregulated DEGs.

GO enrichment analysis was performed to further understand the functional implications of the DEGs identified in the RSC96 cells treated with H_2_O_2_ and MeCbl. The upregulated genes in the MeCbl-treated group were significantly enriched in biological processes (BP) related to mitochondrial function and protein synthesis. These processes included mitochondrial respiratory chain complex assembly (GO:0033108), oxidative phosphorylation (GO:0006119), ribonucleoprotein complex biogenesis (GO:0022613), and ribosome biogenesis (GO:0042254). Notably, genes involved in the aerobic electron transport chain (GO:0019646) and the establishment of protein localization to mitochondria (GO:0072655) were also upregulated. In terms of cellular components (CC), the upregulated genes were primarily associated with mitochondrial protein-containing complexes (GO:0098798), ribosomal subunits (GO:0044391), and the inner mitochondrial membrane protein complex (GO:0098800) as well as the mitochondrial matrix (GO:0005759) and respiratory chain complex I (GO:0005747). At the molecular function (MF) level, these genes were enriched in activities such as the structural constituent of the ribosome (GO:0003735), rRNA binding (GO:0019843), electron transfer activity (GO:0009055), proton-transporting ATP synthase activity (GO:0046933), antioxidant activity (GO:0016209), and iron-sulfur cluster binding (GO:0051536) (Figure 3A).

On the other hand, the downregulated genes in the MeCbl-treated cells were associated with various biological processes related to cellular stress and the regulation of complex disassembly. These processes included the regulation of protein-containing complex disassembly (GO:0043244), chromosome segregation (GO:0007059), cellular responses to stress and radiation (GO:0080135, GO:0071478) as well as the negative regulation of gene expression and epigenetic processes (GO:0045814). Additionally, several downregulated genes were linked to cellular components such as the nuclear chromosome (GO:0000228), nuclear speck (GO:0016607), condensed chromosome (GO:0000793), midbody (GO:0030496), DNA repair complexes (GO:1990391), and sites of DNA damage (GO:0090734). Molecular function analysis revealed that these genes were involved in histone modifying activity (GO:0140993), ATP-dependent processes, acting on DNA (GO:0008094), small GTPase binding (GO:0031267), ATP-dependent chromatin remodeling activity (GO:0140658), histone H3 demethylase activity (GO:0141052), and dystroglycan binding (GO:0002162) (Figure 3B).

These GO enrichment results suggest that MeCbl treatment modulates key cellular pathways, particularly those involved in mitochondrial function and stress responses, which may contribute to its protective effects against oxidative damage.

### 3.4. KEGG Analysis of DEGs

To gain further insight into the functional pathways affected by oxidative stress and MeCbl treatment, KEGG pathway enrichment analysis was conducted on the DEGs in RSC96 cells following H_2_O_2_ exposure and MeCbl treatment. The upregulated DEGs in the MeCbl group were primarily enriched in pathways associated with cellular metabolism and neurodegenerative diseases. These included the ribosome pathway (rno03010), oxidative phosphorylation (rno00190), and pathways related to neurodegenerative diseases such as Parkinson’s disease and Huntington’s disease (rno05012, rno05016). Additionally, genes involved in chemical carcinogenesis through ROS production (rno05208, Figure S1), thermogenesis (rno04714), and amyotrophic lateral sclerosis (rno05014) were also significantly enriched (Figure 3C). These results suggest that MeCbl may enhance mitochondrial function and protect against oxidative stress by modulating critical cellular and metabolic pathways.

In contrast, the downregulated DEGs were enriched in pathways associated with cell proliferation, cell adhesion, and cancer progression. These included pathways related to the cell cycle (rno04110), focal adhesion (rno04510), and proteoglycans in cancer (rno05205). Other significantly enriched pathways included adherens junctions (rno04520), platinum drug resistance (rno01524), regulation of the actin cytoskeleton (rno04810), and the TGF-beta signaling pathway (rno04350) (Figure 3D). These findings indicate that MeCbl treatment may influence cellular processes such as adhesion and cell cycle regulation, potentially contributing to its protective effects under oxidative stress conditions.

Together, these KEGG pathway analyses highlight the complex molecular mechanisms through which MeCbl mediates its protective effects in RSC96 cells, particularly through the modulation of oxidative stress responses, mitochondrial function, and cell survival pathways.

### 3.5. GO-Enriched Gene Sets and PPI Networks

**Figure 4:**
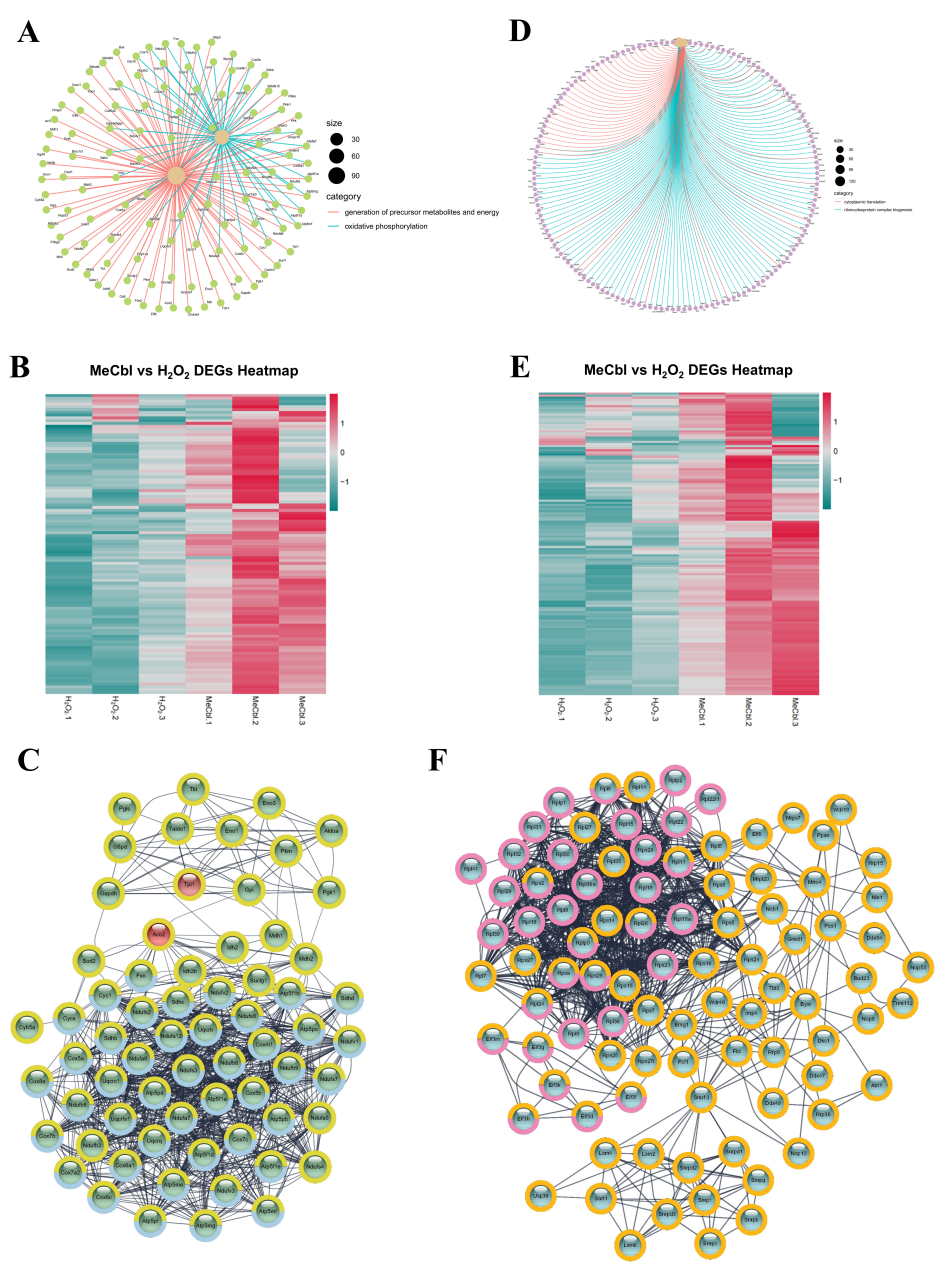
GO-enriched upregulated gene sets and PPI networks. (**A**,**B**) Generation of precursor metabolites and energy and oxidative phosphorylation: an integrated display combining a gene-concept network with the corresponding expression heatmap. (**C**) PPI network for genes in the generation of precursor metabolites and energy and oxidative phosphorylation. Yellow border: generation of precursor metabolites and energy pathways. Blue border: generation of precursor metabolites and energy pathways. (**D**,**E**) Cytoplasmic translation and ribonucleoprotein complex biogenesis: a combined chord diagram and expression heatmap. (**F**) PPI network for cytoplasmic translation and ribonucleoprotein complex biogenesis. Orange border: ribonucleoprotein complex biogenesis pathway. Purple border: cytoplasmic translation pathway.

GO enrichment analysis coupled with expression visualization revealed coherent transcriptional programs modulated by MeCbl under oxidative stress. For upregulated DEGs, the integrated gene-concept network and heatmaps demonstrated a coordinated upregulation of pathways involved in the generation of precursor metabolites and energy as well as oxidative phosphorylation (Figure 4A,B). Genes mapping to these terms were consistently elevated in the MeCbl group compared with the H_2_O_2_, indicating reinforcement of mitochondrial bioenergetic function. The corresponding PPI graph resolved dense modules representing respiratory chain assemblies and ATP synthase, with highly connected nodes forming interaction cores, supporting the recovery of electron transport and ATP production (Figure 4C).

A second upregulated program encompassed cytoplasmic translation and ribonucleoprotein complex biogenesis (Figure 4D,E). The chord diagram highlighted extensive gene sharing among these processes, and the companion heatmap showed the coordinated upregulation of both the ribosomal constituents and biogenesis factors. The PPI network displayed large, highly interconnected clusters representing ribosomal subunits and assembly machinery, together indicating the reinforcement of translational capacity alongside metabolic recovery (Figure 4F).

**Figure 5:**
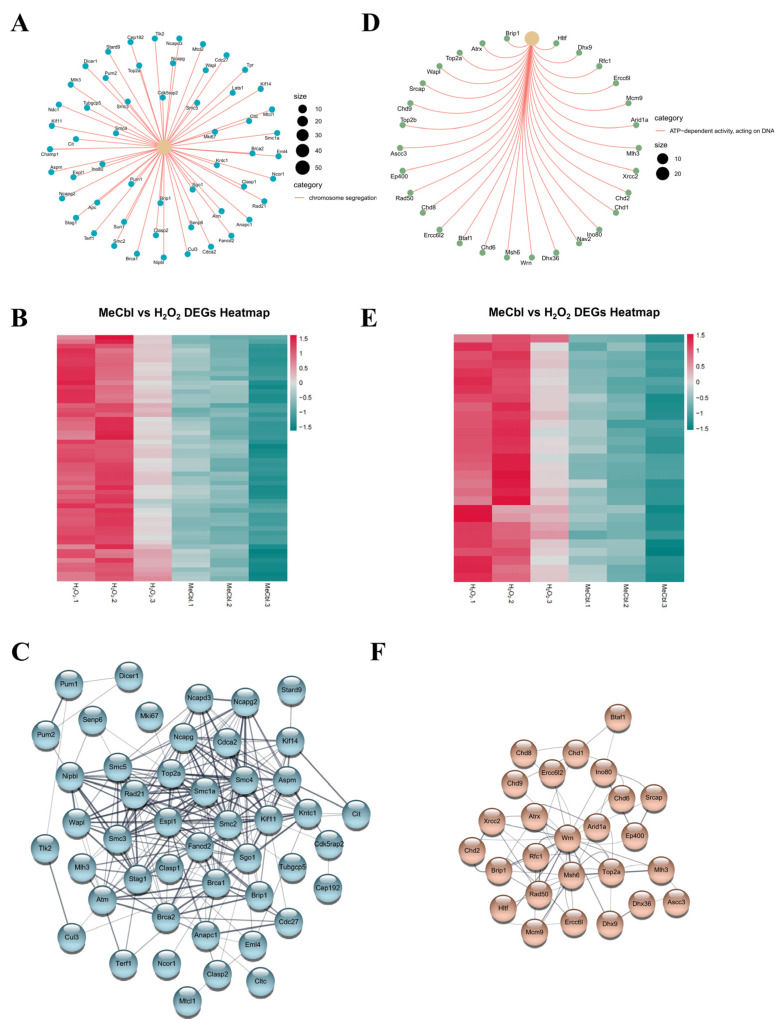
GO-enriched downregulated gene sets and PPI networks. (**A**,**B**) Chromosome segregation: integrated gene-concept network and expression heatmap for downregulated DEGs. (**C**) PPI network for genes in chromosome segregation retrieved from STRING. (**D**,**E**) ATP-dependent activity, acting on DNA: combined chord diagram and expression heatmap for downregulated DEGs. (**F**) PPI network for genes in ATP-dependent activity, acting on DNA.

Among the downregulated programs, genes involved in chromosome segregation genes formed a tightly connected network and were consistently suppressed in MeCbl-treated cells (Figure 5A,B). The STRING-derived PPI network revealed modules enriched for mitotic spindle and kinetochore-related interactions, suggesting the attenuation of cell-cycle progression by MeCbl (Figure 5C). Likewise, genes annotated for ATP-dependent activity, acting on DNA, showed broad suppression (Figure 5D,E), and the associated PPI map contained interaction clusters characteristic of ATP-dependent chromatin remodeling and DNA maintenance complexes (Figure 5F).

Collectively, these analyses indicate a shift from proliferation- and DNA-transaction-oriented programs toward mitochondrial energy metabolism and protein homeostasis support after MeCbl exposure. The network topology aligns with imaging-based evidence of improved mitochondrial morphology and function under MeCbl treatment.

### 3.6. Network Pharmacology Analysis

**Figure 6:**
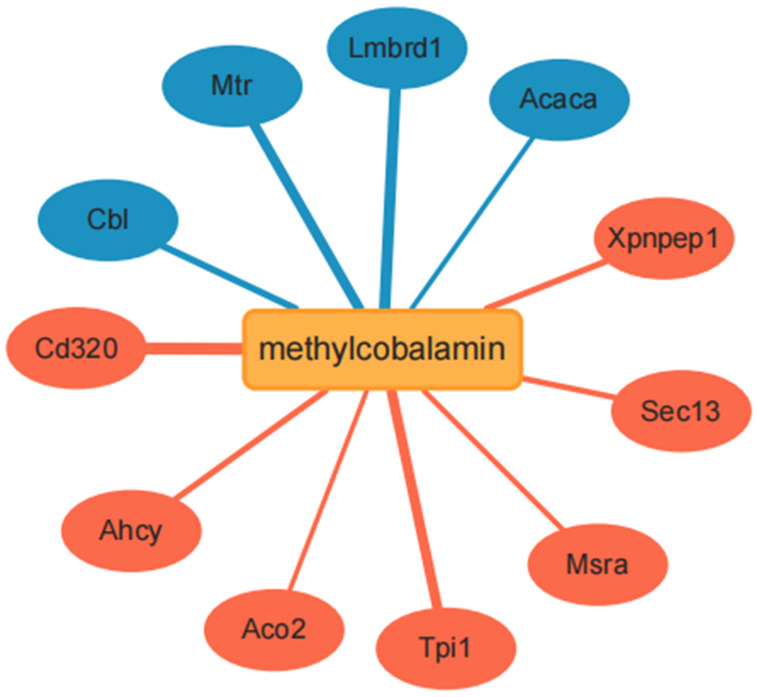
Chemical-gene interaction network retrieved from STITCH showing MeCbl-linked targets. Upregulated genes (Cd320, Ahcy, Aco2, Tpi1, Msra, Sec13, and Xpnpep1) and downregulated genes (Cbl, Mtr, Lmbrd1, and Acaca) are displayed as nodes.

To investigate the potential molecular targets of MeCbl, we used the STITCH database to retrieve genes that were differentially expressed following MeCbl treatment. The analysis identified several upregulated genes including Cd320, Ahcy, Aco2, Tpi1, Msra, Sec13, and Xpnpep1 as well as downregulated genes such as Cbl, Mtr, Lmbrd1, and Acaca (Figure 6). Among these genes, Tpi1 and Aco2 are also distinguished in Figure 4C. These genes were then subjected to GO enrichment analysis, which highlighted significant BP associated with cellular metabolism and oxidative stress responses (Table 3). The upregulated genes were enriched in processes related to glycolysis, NADH regeneration, ribonucleoside catabolism, and cellular respiration. Notably, several processes linked to cellular stress responses, such as the cellular response to oxidative and chemical stress, were also significantly enriched. These findings suggest that MeCbl plays a key role in regulating metabolic processes and enhancing the cellular response to oxidative stress, likely through the modulation of energy production pathways and mitochondrial function.

In addition, the network pharmacology approach provided a broader understanding of the molecular interactions and biological processes potentially influenced by MeCbl. By integrating transcriptomic data with network analysis, we identified key molecular targets and pathways, such as Tpi1-related NADH regeneration, canonical glycolysis, NADH metabolic process, and Aco2-related energy derivation by the oxidation of organic compounds, which are likely involved in MeCbl’s therapeutic effects under oxidative stress conditions. These insights lay the foundation for further experimental validation of MeCbl’s molecular targets and its potential for therapeutic application in oxidative stress-related diseases.

This combined transcriptomic and network pharmacology analysis underscores the multifaceted role of MeCbl in regulating cellular metabolism, oxidative stress responses, and mitochondrial function, offering a comprehensive view of its molecular action in RSC96 cells.

## 4. Discussion

MeCbl has long been used for peripheral neuropathy, with preclinical and clinical evidence suggesting benefits for axonal growth, myelin integrity, and symptom control, but its specific mechanisms remain incompletely understood [18,19,20]. Some studies have shown that MeCbl can improve cell function in different ways under oxidative stress [21,22,23]. For example, An et al. reported that the Nrf2/HO-1 pathway is activated by MeCbl to protect melanocytes from oxidative stress [23]. In our dataset, KEGG enrichment analysis revealed significant upregulation of the Nrf2 downstream genes NQO1, AKR, and HO-1 in the “chemical carcinogenesis-reactive oxygen species” pathway. Additionally, products related to mitochondrial function and oxidative stress were also notably elevated. These findings extend beyond the conventional understanding of MeCbl as a simple neurotrophic factor, revealing its role as a mitochondrial protective agent and a regulator of cellular stress pathways. Supported by in-depth imaging and molecular evidence, our work provides new insights into the functional regulation of Schwann cells in the context of peripheral neuropathy caused by conditions such as diabetes, herpes virus infection, and physical trauma.

Transcriptome and network pharmacology integration provided mechanistic insights into the observed phenotypes, thereby elucidating their molecular basis. GO enrichment analysis revealed that the “generation of precursor metabolites and energy” pathway was significantly upregulated, with subsequent PPI network analysis identifying two key genes, Tpi1 and Aco2. These genes were similarly upregulated in the network pharmacology analysis of MeCbl, reinforcing their potential significance in the therapeutic effects observed. In contrast, GO enrichment analysis also identified several downregulated pathways, notably those related to “chromosome segregation” and “ATP-dependent activity, acting on DNA”. These pathways are typically associated with cell division and DNA repair processes, which are often compromised during oxidative stress. The downregulation of these pathways following H_2_O_2_-induced oxidative stress and subsequent MeCbl treatment suggests that MeCbl may help mitigate oxidative damage by stabilizing cellular functions and reducing the disruption of essential processes such as DNA replication and repair. This could further explain the protective effects of MeCbl in maintaining cellular integrity under stress conditions. Moreover, the network pharmacology analysis of MeCbl identified the downregulation of four genes (Cbl, Mtr, Lmbrd1, and Acaca), each of which may contribute to the observed effects. The downregulation of these genes in the MeCbl-treated group may reflect a coordinated modulation of the metabolic and repair pathways, contributing to the overall protective effect against oxidative stress and promoting cellular recovery.

Mitochondria serve as central regulators of Schwann cells’ physiology, supporting both myelin lipid synthesis and maintaining axon–glia metabolic coupling [24,25]. Their dynamic process (fission-fusion) directly influences bioenergetics, calcium handling, and cell fate. Disruption of this balance, usually manifested as DRP 1-mediated excessive fission coupled with impaired MFN 2- or OPA 1-dependent fusion, leads to mitochondrial fragmentation and functional decline, hallmarks of oxidative stress in neural cells [26,27]. Although Schwann cells can activate antioxidant responses to counter oxidative challenges, sustained ROS weaken these defenses and disrupt organelle networks. By reconstructing a reticular mitochondrial structure and enhancing oxidative phosphorylation-related gene expression, MeCbl in this model appears to disrupt the positive feedback loop between ROS production and mitochondrial dysfunction. This effect aligns mechanistically with its role in one-carbon/redox metabolism and supports prior reports that B12 supplementation improves neural repair.

Several limitations must be acknowledged. First, our results were derived from an in vitro RSC96 model and a single oxidative stress paradigm. To define the therapeutic threshold, systematic dose–response and time-course profiles for H_2_O_2_ and MeCbl should be established. Additionally, a milder and more gradual stress model (such as metabolic stress) should be employed to validate the universality of the findings in this study. Second, although ultrastructural analysis correlates with transcriptional readouts, establishing causality requires direct functional assays including measurements of DRP1 phosphorylation, MFN2 and OPA1 abundance, and key mitochondrial autophagy markers such as PINK1/Parkin, LC3-II, and p62. Third, the candidate mediators identified in this study (Cd320, Ahcy, Tpi 1, Aco 2, Msra) require validation of their target mechanisms through perturbation and recovery experiments. Finally, translational research should establish an in vivo peripheral neuropathy model to elucidate the relationship between MeCbl exposure and Schwann cell mitochondrial integrity, inflammatory status, remyelination, and functional recovery.

## 5. Conclusions

To sum up, MeCbl mitigates H_2_O_2_-induced oxidative injury in Schwann cells by restoring a reticular mitochondrial network and reversing fragmentation. Concordant transcriptomic and network pharmacology analyses implicate the reinforcement of mitochondrial organization, quality control, and antioxidant defenses as plausible mechanisms. These findings nominate MeCbl as a rational adjunct for oxidative stress-driven peripheral neuropathology and outline testable molecular axes for in vivo validation.

## Figures and Tables

**Table 1:** The top ten up- or downregulated DEGs.

Threshold	Gene	*p*-adj	Log2FC	Threshold	Gene	*p*-adj	Log2FC
Upregulated	*Capns1*	7.76 × 10^−130^	5.58	Downregulated	*Brca2*	3.79 × 10^−5^	−1.55
	*LOC103694876*	1.88 × 10^−37^	3.74		*Rnf213*	3.79 × 10^−5^	−0.88
	*LOC100912427*	1.85 × 10^−9^	1.60		*Prpf4b*	4.24 × 10^−5^	−1.51
	*AABR07044509.1*	1.43 × 10^−7^	1.39		*Frs2*	5.69 × 10^−5^	−0.89
	*Cox5b*	1.42 × 10^−5^	1.17		*Usp32*	6.08 × 10^−5^	−1.10
	*Psmb7*	1.74 × 10^−5^	0.80		*Nipbl*	6.45 × 10^−5^	−1.07
	*AABR07050545.1*	1.74 × 10^−5^	1.26		*Fbn1*	7.07 × 10^−5^	−0.78
	*AABR07026997.1*	2.38 × 10^−5^	1.53		*Apc*	8.28 × 10^−5^	−0.99
	*Rplp0*	3.79 × 10^−5^	1.02		*Ogt*	9.60 × 10^−5^	−1.25
	*LOC108353446*	3.79 × 10^−5^	1.43		*Rock1*	1.17 × 10^−4^	−1.13

*p*-adj, *p*-adjusted; FC, fold change.

**Table 2:** Description of the top ten up- and downregulated genes with the most significant differences.

Upregulated	Molecular Function	Downregulated	Molecular Function
Capns1	Calcium ion binding	Brca2	Single-stranded DNA binding
LOC103694876	Chromatin binding	Rnf213	Zinc ion binding
LOC100912427	Na	Prpf4b	ATP binding
AABR07044509.1	Na	Frs2	Transmembrane receptor protein tyrosine kinase adaptor activity
Cox5b	Protein binding	Usp32	Cysteine-type deubiquitinase activity
Psmb7	Endopeptidase/peptidase activity	Nipbl	Chromatin binding
AABR07050545.1	Na	Fbn1	Structural constituent of extracellular matrix
AABR07026997.1	Structural constituent of ribosome	Apc	Beta-catenin binding
Rplp0	Peptide binding	Ogt	Hexosyltransferase activity
LOC108353446	Na	Rock1	Small GTPase binding

**Table 3:** GO enrichment analysis of upregulated DEGs.

ID	Description	Fold Enrichment	Gene ID
GO:0019682	Glyceraldehyde-3-phosphate metabolic process	156.31	*Tpi1*
GO:0042454	Ribonucleoside catabolic process	132.26	*Ahcy*
GO:0006735	NADH regeneration	114.62	*Tpi1*
GO:0061621	Canonical glycolysis	114.62	*Tpi1*
GO:0019677	NAD catabolic process	90.49	*Tpi1*
GO:1901658	Glycosyl compound catabolic process	68.77	*Ahcy*
GO:0006734	NADH metabolic process	50.57	*Tpi1*
GO:0032527	Protein exit from endoplasmic reticulum	39.99	*Sec13*
GO:0045333	Cellular respiration	6.91	*Aco2*
GO:0034599	Cellular response to oxidative stress	5.56	*Msra*
GO:0015980	Energy derivation by oxidation of organic compounds	4.86	*Aco2*
GO:0062197	Cellular response to chemical stress	4.37	*Msra*

## Data Availability

Data are contained within the article.

## Supplementary Materials

The following supporting information can be downloaded at: https://www.mdpi.com/article/10.3390/biomedicines13102565/s1, Video S1: Control group; Video S2: H_2_O_2_ group; Video S3: MeCbl group. Figure S1: KEGG pathway map of chemical carcinogenesis-reactive oxygen species.
