# Supplementary material for: Effects of Methylcobalamin on Mitochondrial Alterations in Schwann Cells Under Oxidative Stress
Source: Biomedicines. 2025 Oct 21;13(10):2565. doi: 10.3390/biomedicines13102565 (PMC12561138; doi:10.3390/biomedicines13102565)
Supplement: Supplementary file 1 [file biomedicines-13-02565-s001.zip › Supplementary materials.pdf]

Supplementary materials

Effects of Methylcobalamin on Mitochondrial Alterations in Schwann Cells under Oxidative Stress

Qicheng Li [1,2,†], Shiyan Liu [4,†], Lu Zhang [1,2,4,†], Tianze Sun [1,2] and Yuhui Kou [1,2,3,*]

[1]Department of Trauma and Orthopedics, Peking University People's Hospital, Beijing 100044, China;

qicheng.li@pku.edu.cn (Q.L.); LorraineZ0827@163.com (L.Z.); suntianze1997@163.com (T.S.)

[2]Key Laboratory of Trauma and Neural Regeneration, Peking University, Beijing 100044, China

[3]National Center for Trauma Medicine, Beijing 100044, China

[4]Department of Physiology, School of Basic Medical Sciences, Shenzhen University, Shenzhen 518060, China;

2100243061@email.szu.edu.cn (S.L.)

[*] Correspondence: yuhuikou@bjmu.edu.cn

[†]These authors contributed equally to this work.

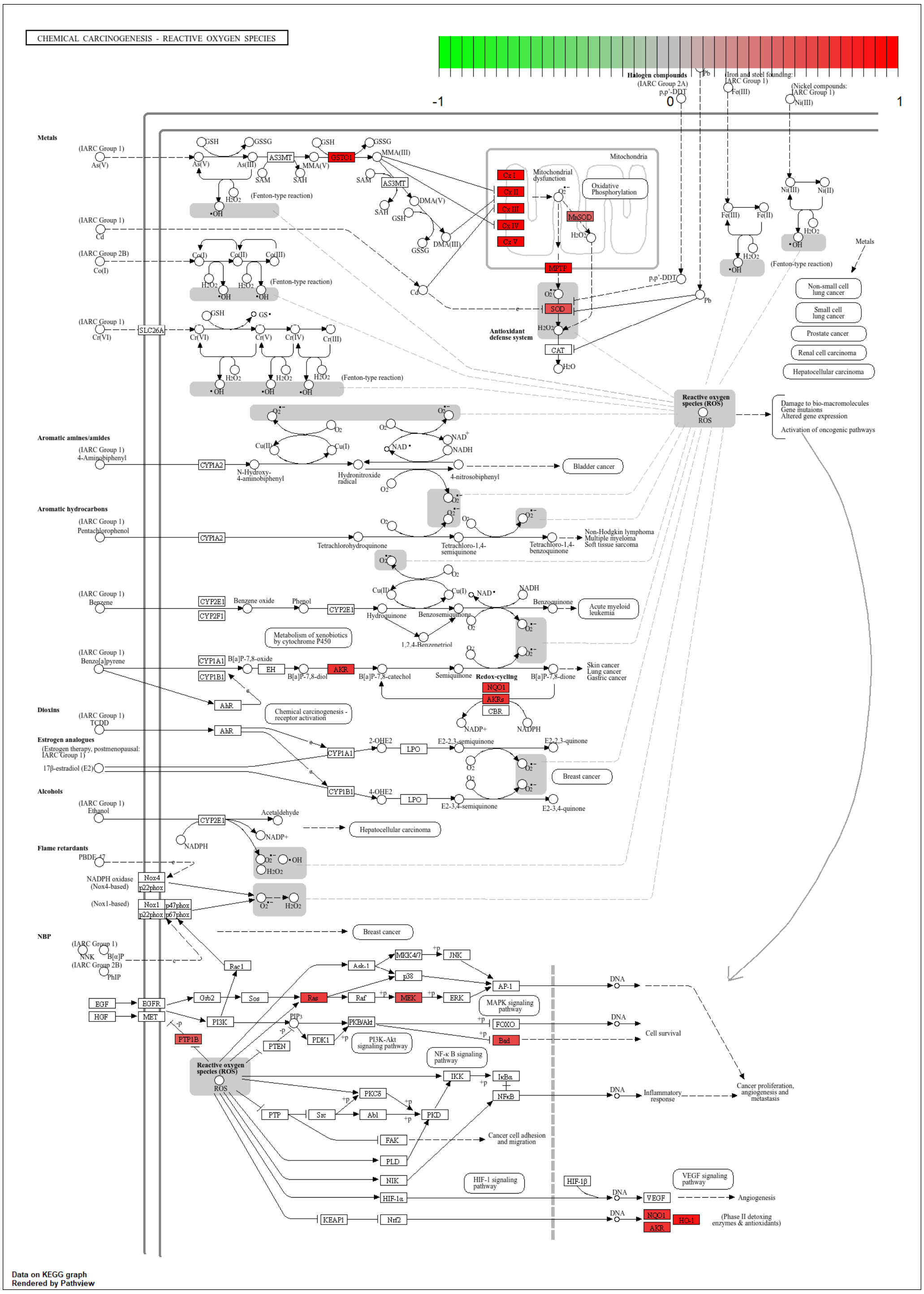

Figure S1. KEGG pathway map of chemical carcinogenesis-reactive oxygen species.
